# Beyond conventional treatments: cutting-edge strategies for ascites in cirrhosis

**DOI:** 10.3389/fmed.2026.1918993

**Published:** 2026-09-28

**Authors:** Weimiao Lin, Wenjin Fan, Lei Xu, Lihong Mao

**Affiliations:** 1 Department of Gastroenterology, The First Affiliated Hospital of Ningbo University, Ningbo, China; 2 Endoscopy Center, The First Affiliated Hospital of Ningbo University, Ningbo, China

**Keywords:** ascites, cirrhosis, gut microbiota, regenerative medicine, SGLT2 inhibitors, vasopressin analogues

## Abstract

Ascites is a prevalent complication in cases of decompensated liver cirrhosis, having a considerable effect on the quality of life and prognosis of patients. Current treatment methods of ascites in cirrhosis primarily include conventional diuretics, paracentesis, albumin infusion, transjugular intrahepatic portosystemic shunt (TIPS), and liver transplantation. However, these approaches are associated with certain limitations and risks. In recent years, novel therapeutic strategies targeting distinct pathophysiological pathways of ascites have continued to emerge, including sodium–glucose cotransporter 2 (SGLT2) inhibitors, vasopressin analogues, regenerative medicine, and gut microbiota modulation. The present review focuses on emerging pharmacological strategies for ascites in cirrhosis, systematically summarizes their mechanisms of action, and discusses their efficacy, safety, and limitations, while also outlining future research directions.

## 1 Introduction

Ascites is a major issue that arises from liver cirrhosis. The development of ascites involves a complex pathophysiological process, primarily driven by portal hypertension and sodium retention (1). Around 50%–60% of individuals diagnosed with liver cirrhosis will experience ascites within a decade, and the three-year mortality rate can reach 50% (2). In addition, complications such as spontaneous bacterial peritonitis, hepatorenal syndrome (HRS), and refractory ascites can further increase mortality rate. For this reason, early diagnosis and management of ascites are critical for enhancing both patient survival and quality of life.

Current management of ascites in cirrhosis relies on loop diuretics and aldosterone antagonists, which promote diuresis by altering renal reabsorption of water and electrolytes. However, excessive or inappropriate use of these agents may result in negative effects like electrolyte imbalances, renal impairment, and hypotension (3). Additionally, prolonged use of traditional diuretics might cause diuretic resistance, which could be related to modifications in neurohormonal signaling, reduced drug secretion by the tubules, and weakened sodium-chloride sensing (4). When conventional diuretics fail to control ascites, additional interventions are required. These might encompass therapeutic paracentesis, albumin infusion, transjugular intrahepatic portosystemic shunt (TIPS), or even liver transplantation (1, 5). However, each of these approaches carries its own risks of complications. For instance, repeated therapeutic paracentesis increases the need for hospitalization and raises the risk of intra-abdominal infection. TIPS is frequently associated with hepatic encephalopathy. Therefore, a major clinical challenge lies in maintaining effective diuresis while minimizing treatment-related adverse effects.

Therefore, the present review focuses on recent advances in emerging pharmacological therapies for ascites in cirrhosis. Based on the multiple pathophysiological mechanisms underlying ascites formation, SGLT2 inhibitors, vasopressin analogues, regenerative medicine, and gut microbiota modulation were selected as the core topics for discussion. Aiming to provide a beneficial reference, this review focuses on novel clinical therapeutic strategies for ascites in cirrhosis and outlines potential future research directions.

## 2 Pathogenesis of ascites in cirrhosis

The development of ascites in cirrhosis is a multifaceted pathophysiological process that includes the liver, the circulatory system, and the kidneys. Structural changes in the cirrhotic liver lead to impaired portal venous return, resulting in portal hypertension. The marked elevation in portal pressure increases mesenteric capillary hydrostatic pressure, thereby promoting fluid leakage into the peritoneal cavity. Second, remodeling of the intrahepatic architecture leads to hepatic venous outflow obstruction and increased hepatic lymph production. When the thoracic duct's drainage capacity is surpassed, lymph leaks from the liver capsule into the peritoneal cavity. In addition, hepatic synthetic function is markedly impaired in patients with cirrhosis, resulting in reduced albumin production and decreased plasma oncotic pressure, which promotes fluid extravasation from the intravascular compartment (6). Elevated portal pressure increases nitric oxide (NO) production, leading to splanchnic arterial vasodilation, which in turn augments portal venous inflow and further exacerbates portal hypertension (1). A significant volume of blood accumulates in the expanded splanchnic vascular region, causing a relative shortage in effective arterial blood volume, which in turn activates the renin–angiotensin–aldosterone system (RAAS), the sympathetic nervous system (SNS), and promotes the secretion of antidiuretic hormone (ADH) (7). Activation of the RAAS and SNS promotes renal sodium reabsorption, while increased secretion of ADH enhances renal water reabsorption. In addition, cirrhosis can trigger gut microbiota dysbiosis via the gut-liver axis's bidirectional regulation, exacerbating systemic inflammation and furthering cirrhosis development (8). Collectively, these mechanisms lead to the formation of ascites (Figure 1).

**Figure 1:**
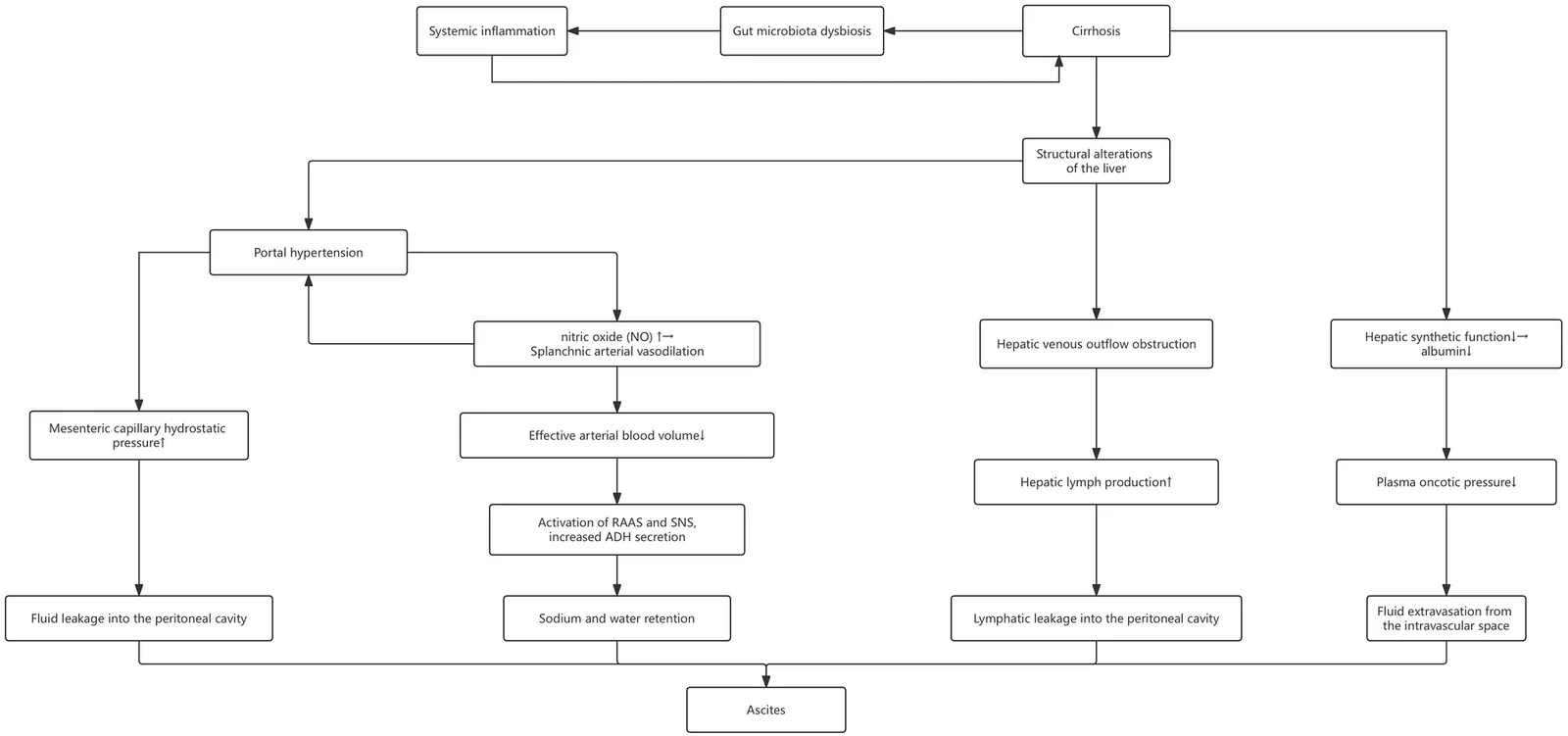
The pathogenesis of ascites in cirrhosis. (RAAS: renin–angiotensin–aldosterone system; SNS: sympathetic nervous system; ADH: antidiuretic hormone).

## 3 SGLT2 inhibitors: a novel diuretic strategy targeting renal sodium and water excretion

### 3.1 Mechanism of SGLT2 inhibitors

SGLT2 is a protein transporter found in the proximal renal tubules, tasked with reabsorbing most of the glucose filtered by the kidneys (9). SGLT2 inhibitors were initially used as antidiabetic medications, which can reduce glucose levels in the blood by preventing SGLT2 from reabsorbing glucose, thereby promoting urinary glucose excretion. In recent years, studies have revealed that SGLT2 inhibitors also offer protective benefits for both cardiovascular and renal systems (10). SGLT2 inhibitors block SGLT2, resulting in increased sodium delivery along the nephron, inhibition of sodium reabsorption, enhanced natriuresis, and improved urinary output. In addition, by increasing sodium delivery to the macula densa, they suppress renin secretion and attenuate RAAS activity (11–13). These mechanisms directly target the “renal sodium and water retention” component in the pathogenesis of ascites in cirrhosis. Importantly, they do not rely solely on the aldosterone pathway, as conventional diuretics do, which support its potential application in patients with diuretic resistance from a theoretical perspective.

### 3.2 Therapeutic effect in ascites

The evidence available on SGLT2 inhibitors for managing ascites in cirrhosis is largely from small clinical studies. Multiple case reports have demonstrated that in patients with cirrhosis and concurrent diabetes, SGLT2 inhibitors can improve fluid retention, reduce ascites, and allow for dose reduction of conventional diuretics, thereby decreasing the incidence of their adverse effects (14, 15). Dapagliflozin and empagliflozin are commonly used SGLT2 inhibitors. Multiple clinical studies have demonstrated that both dapagliflozin and empagliflozin can control ascites and increase urinary sodium excretion, and that they also exert certain therapeutic effects on refractory ascites (16–18). However, these studies are restricted by having small sample sizes and limited follow-up durations, and are currently insufficient to support routine clinical recommendation. By contrast, a large-scale propensity score-matched cohort study demonstrated that SGLT2 inhibitors greatly decreased the rate of serious hepatic incidents, including the development of esophageal or gastric varices, hyponatremia, ascites, or death from any cause (19). However, this study was retrospective in design and could not exclude residual confounding. Moreover, the composite endpoint included non-ascites-related events and was not specifically designed for patients with ascites.

Although SGLT2 inhibitors increase urinary sodium excretion, they may paradoxically improve hyponatremia to some extent. This effect may be attributed to mechanisms such as SGLT2 inhibitor-induced osmotic diuresis (20). Furthermore, SGLT2 inhibitors have minimal impact on serum potassium levels (21).

### 3.3 Limitations and challenges

At present, there is limited safety data on SGLT2 inhibitors for patients with cirrhosis. Some studies suggest a potential increased risk of adverse events, such as infections and acute renal failure (16), while others found no association between the use of SGLT2 inhibitors and bacteremia, identifying male sex and cirrhosis as potential risk factors for infections (22). Although according to some studies, SGLT2 inhibitors may help in slowing the decline of kidney function through mechanisms such as preserving estimated glomerular filtration rate (eGFR), reducing intraglomerular pressure, and lowering uric acid levels (23–26), these studies were not conducted in patients with end-stage liver disease. The adverse effects of SGLT2 inhibitors may be more pronounced in this population. What's more, several case reports and meta-analyses have raised concerns about an elevated risk of diabetic ketoacidosis with the use of SGLT2 inhibitors (23, 27, 28). This could be due to a swift rise in glucose excretion through urine, which results in decreased plasma insulin levels, diminished glucose utilization, and increased fat breakdown (28).

The application of SGLT2 inhibitors for managing ascites in cirrhosis is still under investigation and is not recommended as a routine therapy. Based on current evidence, SGLT2 inhibitors might be an individualized experimental choice for patients with type 2 diabetes mellitus who do not respond well to traditional diuretics, but close monitoring of ketosis, infection, and renal function is required. Table 1 summarizes the potential benefits and risks of SGLT2 inhibitors in clinical studies for treating ascites in cirrhosis.

**Table 1:** Clinical studies of SGLT2 inhibitors for ascites in cirrhosis.

Study/ Type of study	Number and characteristics of study population	Study groups	Benefits	Risks
Singh, 2025 (16)/ double-blind RCT	40 cirrhotic patients with recurrent ascites	Dapagliflozin + SMT (*n* = 20), placebo + SMT (*n* = 20)	Improvement in ascites control; increasement of urinary sodium excretion	Increased incidence of acute kidney injury and infections
Kalambokis, 2024 (17)/ single-arm trial	14 cirrhotic patients with refractory ascites	Empagliflozin (*n* = 14)	Improvement in ascites control; increasement of urinary sodium excretion	No severe adverse events
Hu, 2025 (18)/ single-arm trial	14 patients with refractory ascites and decompensated cirrhosis	Empagliflozin (*n* = 14)	Improvement in ascites control	One episode of acute kidney injury; one episode of urinary tract infection
Abu-Hammour, 2025 (19)/ retrospective cohort study	10,660 cirrhotic patients	SGLT-2 inhibitors + SMT (*n* = 5,330); SMT (*n* = 5,330)	Improvement in ascites control, hepatorenal syndrome, spontaneous bacterial peritonitis, variceal bleeding; decreased rate of serious liver events and hospitalizations	/

RCT, randomized controlled trial; SMT, standard medical therapy.

## 4 Vasopressin analogues: targeting splanchnic vasodilation and portal hypertension

### 4.1 Terlipressin

#### 4.1.1 Mechanism of terlipressin

Terlipressin is a synthetic vasopressin analogue with potent vasoconstrictive effects on the splanchnic circulation. Portal hypertension is a primary factor contributing to the development of ascites in cirrhosis. By selectively activating V1a receptors on vascular smooth muscle cells, terlipressin induces splanchnic vasoconstriction, thereby reducing portal venous inflow and ameliorating portal hypertension (29). Additionally, terlipressin helps attenuate the activation of RAAS, which further reduces sodium and fluid retention (30). Furthermore, terlipressin has been shown to downregulate the expression of aquaporin-2 (AQP2), a water channel protein located in the renal collecting ducts that promotes water reabsorption (31). These mechanisms underscore the potential role of terlipressin in the treatment of ascites associated with cirrhosis.

#### 4.1.2 Therapeutic effect in ascites

Small-scale clinical studies have found that terlipressin can enhance renal function and cause natriuresis even in cirrhotic patients with ascites, even if they do not have HRS (32). Another meta-analysis found that terlipressin is capable of improving hemodynamics, reducing the severity of ascites, and enhancing renal function in cirrhosis patients with ascites who do not have HRS, and may prevent post-paracentesis circulatory dysfunction (33). Collectively, these results indicate that terlipressin could be a promising new treatment option for ascites in cirrhosis. Furthermore, the use of terlipressin alongside albumin and diuretics has a combined beneficial impact on patients with refractory ascites. The combination of terlipressin, diuretics, and albumin improves outcomes and reduces ascites severity for patients with refractory ascites who do not have HRS (34). Currently, existing guidelines recommend that terlipressin should be an option for cirrhosis patients with severe or refractory ascites when diuretics do not work or are not well tolerated due to side effects (35).

Terlipressin is currently administered primarily via continuous intravenous infusion or intravenous bolus injection. Multiple clinical studies have found that continuous terlipressin infusion can effectively reduce ascites and the frequency of paracentesis in patients with cirrhosis, while also lowering serum creatinine, increasing urinary sodium excretion, and improving quality of life (36, 37). Moreover, prolonged continuous administration of terlipressin can greatly improve cardiac reserve and reduce the hyperdynamic circulatory condition in those suffering from decompensated cirrhosis (38). Currently, no large-scale clinical trials have specifically evaluated terlipressin in cirrhosis patients with ascites. Based on the treatment approach for HRS, it is recommended in guidelines to administer an initial 1 mg dose every 12 h through a continuous intravenous infusion to control ascites in cirrhosis (35).

#### 4.1.3 Adverse effect

The most frequent and notable adverse effect of terlipressin is hyponatremia, with the incidence of severe hyponatremia (defined as sodium levels ≤125 mmol/L) occurring in approximately 33% to 44% of patients. A higher baseline serum sodium level and prolonged duration of terlipressin therapy are correlated with a greater likelihood of developing severe hyponatremia (39, 40). In addition, owing to its potent vasoconstrictive effects, terlipressin has been associated with other adverse effects, including cutaneous ischemic necrosis, severe cardiovascular adverse events, gastrointestinal spasm and ischemia, and diarrhea (41, 42). However, these complications do not appear to increase patient mortality and are generally reversible upon discontinuation of the drug and appropriate clinical intervention (30, 43). Table 2 summarizes the potential benefits and risks of terlipressin in clinical studies for treating ascites in cirrhosis.

**Table 2:** Clinical studies of terlipressin for ascites in cirrhosis.

Study/ Type of study	Number and characteristics of study population	Study groups	Benefits	Risks
Krag, 2007 (32)/ partial double-blind RCT	23 alcoholic cirrhotic patients with ascites without HRS	15 patients with nonrefractory ascites: terlipressin (*n* = 11), placebo (*n* = 15) 8 patients with refractory ascites: terlipressin (*n* = 8)	Improvement in renal function; induces natriuresis	No severe adverse events
Fimiani, 2011 (34)/ multicentre, single-arm trial	26 cirrhotic patients with refractory ascites	Terlipressin + diuretics + albumin (*n* = 26)	Improvement in ascites control; increasement of urinary sodium excretion	No severe adverse events
Gow, 2016 (36)/ single-arm trial	5 cirrhotic patients with refractory ascites	CTI (*n* = 5)	Improvement in ascites control; increasement of urinary sodium excretion	No severe adverse events
Ryma, 2024 (37)/ a prospective randomized crossover study	30 cirrhotic patients with refractory ascites	12 weeks of CTI (*n* = 16), 12 weeks of observation prior to CTI (*n* = 14)	Improvement in ascites control and life quality; reduction in serum creatinine; increasement of urinary sodium excretion	No severe adverse events

RCT, randomized controlled trial; CTI, continuous terlipressin infusion.

### 4.2 Novel vasopressin analogues

To overcome the limitations of terlipressin, next-generation vasopressin analogues are currently under development. Castillo et al. developed a novel partial V1a receptor agonist, PHIN-214. Compared to terlipressin, PHIN-214 demonstrates an approximately tenfold higher relative therapeutic index for reducing portal pressure and is associated with a lower risk of dilutional hyponatremia. Furthermore, as it is administered subcutaneously, PHIN-214 avoids the risk of local tissue necrosis, offering a safer and more convenient treatment option for patients with portal hypertension and related complications in cirrhosis (44). Similarly, Fernández-Varo et al. developed FE 204038, a selective V1a-vasopressin (V1a-AVP) receptor agonist, which demonstrated potential therapeutic value in a rat model with cirrhosis and ascites. The researchers discovered that V1a-AVP receptor expression was significantly upregulated in the mesentery of cirrhotic rats, suggesting that V1a-AVP agonists may preferentially induce splanchnic vasoconstriction in cases of cirrhosis. Furthermore, by redistributing intravascular fluid, FE 204038 improved cardiac output and systemic vascular resistance (SVR), leading to significant enhancement of cardiovascular function in cirrhotic rats (45).

In addition, terlipressin activates V2 receptors in the renal collecting ducts, exerting an antidiuretic effect that may partially counteract its diuretic efficacy (46). OCE-205 is a novel, selective partial V1a receptor agonist featuring two distinct functional domains for receptor interaction: one acts as an agonist, while the other functions as an antagonist. This unique design enables effective vascular contraction for therapeutic benefit while minimizing the risk of excessive vasoconstriction, thereby improving safety. In cirrhotic rats with ascites, administration of OCE-205 significantly reduced splanchnic vasodilation and portal hypertension, decreased ascites volume and body weight, and increased urine output. Notably, at therapeutic doses, OCE-205 does not activate V2 receptors, and no instances of hyponatremia have been observed (47).

Currently, a phase II trial is in progress for OCE-205 aimed at treating hepatorenal syndrome-acute kidney injury (HRS-AKI) (48). However, these novel vasopressin analogues remain at the preclinical stage for treating ascites in cirrhosis and have not received approval for any indication. Upcoming initiatives should focus on improving the accuracy and effectiveness of targeted vasoconstrictive treatments. To validate the safety and effectiveness of innovative targeted vasoconstrictors and optimize treatment protocols, large-scale, multicenter clinical trials are also necessary.

## 5 Regenerative medicine: targeting hepatic function restoration

### 5.1 Mechanisms and therapeutic effect of different stem cell therapies

The definitive solution for decompensated cirrhosis remains liver transplantation. However, its application is limited by factors such as donor shortages, high costs, and long-term complications. The development of regenerative medicine has provided new perspectives for treating ascites in cirrhosis, among which stem cell therapy stands out as a particularly robust area of study.

Multiple mechanisms are involved in the therapeutic effects of mesenchymal stem cells (MSCs), including migration to the liver, immunomodulation, hepatic differentiation, and paracrine signaling, thereby suppressing inflammation, reducing hepatocyte apoptosis, promoting hepatocyte regeneration, reversing liver fibrosis, and enhancing hepatic function (49, 50). Several small-scale clinical trials have shown that MSCs therapy can effectively enhance liver function, decrease ascites, and improve the quality of life for cirrhosis patients (51–54). A study by Yao et al. demonstrated that human umbilical cord mesenchymal stem cells (hUCMSCs) facilitate the polarization of M1 macrophages toward the M2 phenotype by activating PPAR*γ* signaling, thereby suppressing inflammation and facilitating the liver tissue repair. Rats with decompensated cirrhosis experienced a marked improvement in liver structure and function due to this intervention. Furthermore, the authors identified an optimal treatment protocol involving weekly administration of hUCMSCs for three weeks during the early stage of decompensated cirrhosis, which provided the greatest therapeutic benefit in lowering mortality and ascites, along with better liver function in a rat model of the disease (55). However, the research conducted by Mohamadnejad et al. revealed that infusing MSCs may not confer benefit in patients with early-stage cirrhosis (56).

Granulocyte colony-stimulating factor (G-CSF) promotes liver regeneration by enhancing the migration of progenitor cells from the bone marrow to the liver and augmenting the endogenous oval cell response (57). Clinical trials have demonstrated that G-CSF therapy can improve ascites, lower the risk of hepatic encephalopathy and infection, enhance quality of life, and may even improve survival (58–61). Moreover, patients with early decompensated cirrhosis and mild-to-moderate ascites, along with healthy bone marrow function at baseline, exhibit a more favorable regenerative response to G-CSF therapy (62). In case of end-stage liver disease, combining G-CSF with MSCs has proven effective in enhancing liver function and reducing ascites in those with cirrhosis (63).

Nonetheless, there have been reports of contradictory findings. Newsome et al. evaluated the efficacy of G-CSF in treating cirrhosis patients, whether combined with hematopoietic stem cell infusion or not (64). It was discovered that G-CSF by itself or in combination with hematopoietic stem cell infusion did not enhance liver function or reduce fibrosis. But the study enrolled participants with compensated cirrhosis. Patients with compensated cirrhosis have a higher percentage of functional liver cells compared to those with decompensated cirrhosis, and therefore possess greater capacity for liver regeneration and reversing fibrosis. Consequently, it is not recommended for patients with compensated cirrhosis to receive stem cell therapy. What's more, Philips et al. conducted a retrospective study and discovered that the improvement in ascites following G-CSF therapy was only transient. Within 6–12 months after treatment, ascites worsened again, and G-CSF treatment was linked to higher rates of sepsis, liver failure, and hepatocellular carcinoma, as well as reduced survival (65).

Conventional stem cell therapy is typically administered via peripheral intravenous infusion, fluoroscopy-guided hepatic artery injection, or abdominal ultrasound-guided portal vein injection. Zheng et al. developed a novel approach using endoscopic ultrasound-guided portal vein injection, which enables precise delivery of stem cells to the liver and appears to be a secure and efficient approach (66). Li et al. developed a percutaneous intrahepatic infusion method using autologous bone marrow nucleated cells (ABMNCs) under B-ultrasound guidance. This technique offers advantages such as minimal invasiveness, reduced pain, no need for hospitalization, rapid recovery, and high patient acceptance, while demonstrating efficacy comparable to that of ABMNC infusion via an implantable port (67). This approach provides a more convenient and accessible clinical option for stem cell therapy in decompensated cirrhosis.

### 5.2 Limitations and challenges

Stem cell therapy has demonstrated potential benefits for cirrhosis. However, the clinical application of stem cell therapy still faces several challenges. These include the need for more precise control over the differentiation of stem cells into functional hepatocytes and ensuring that the differentiated cells exhibit normal hepatic functionality. Additionally, optimal treatment parameters, such as dosage, duration, and timing of administration, remain to be established. Furthermore, the long-term safety and effectiveness of stem cell therapy require validation through larger sample studies and extended follow-up, including thorough evaluation of potential risks such as tumorigenicity.

Currently, stem cell therapy is still under investigation and not recommended for routine clinical use in treating ascites in cirrhosis and should be conducted exclusively within the setting of rigorously designed clinical trials. Table 3 summarizes the potential benefits and risks of MSCs and G-CSF in clinical studies for treating ascites in cirrhosis.

**Table 3:** Clinical studies of MSCs and G-CSF for ascites in cirrhosis.

Study/ Type of study	Number and characteristics of study population	Study groups	Benefits	Risks
Zhang, 2012 (51)/ open-label RCT	45 chronic hepatitis B patients with decompensated cirrhosis	UC-MSCs (*n* = 30), saline (*n* = 15)	Improvement in ascites control and liver function	No severe adverse events
Amin, 2013 (53)/ single-arm trial	20 chronic hepatitis C patients with end-edge cirrhosis	BM-MSCs (*n* = 20)	Improvement in ascites control, CTP score and liver function	No severe adverse events
Wang, 2013 (54)/ single-arm trial	7 patients with primary biliary cirrhosis	UC-MSCs (*n* = 7)	Improvement in ascites control and liver function	No severe adverse events
Mohamadnejad, 2013 (56)/ RCT	27 patients with decompensated cirrhosis	MSCs (*n* = 15), placebo (*n* = 12)	No improvement in liver dysfunction and MELD score	Three patients in the MSCs group died of liver failure
Venkitaraman, 2022 (58)/ double-blind RCT	70 patients with decompensated cirrhosis	G-CSF + SMT (*n* = 35), placebo + SMT (*n* = 35)	Improvement in ascites control and CTP score; decreased rate of encephalopathy episodes and infections	No severe adverse events
De, 2021 (59)/open-label RCT	100 patients with decompensated cirrhosis	G-CSF + SMT (*n* = 50), SMT (*n* = 50)	Higher survival; improvement in ascites control, CTP score and life quality; decreased rate of infections and hospitalizations; lower liver stiffness measurements	No severe adverse events
Verma, 2018 (61)/ open-label RCT	65 patients with decompensated cirrhosis	G-CSF + SMT + GH (*n* = 23), G-CSF + SMT (*n* = 21), SMT (*n* = 21)	Better 12-month TFS; improvement in ascites control, CTP score, MELD score, nutrition and life quality; decreased rate of infections; lower liver stiffness measurements. But the use of GH was not found to have any additional benefit.	No severe adverse events
Anand, 2019 (62)/ double-blind RCT	60 patients with decompensated cirrhosis	G-CSF + EPO (*n* = 30), G-CSF + placebo (*n* = 30)	Higher survival, improvement in ascites control, CTP score and MELD score, decreased rate of encephalopathy episodes and acute kidney injury	One episode of hematemesis in G-CSF + EPO group
Salama, 2014 (63)/ RCT	40 chronic hepatitis C patients with end-edge cirrhosis	G-CSF + MSC (*n* = 20), SMT (*n* = 20)	Improvement in ascites control, liver function and life quality	No severe adverse events
Newsome, 2018 (64)/ multicentre, open-label RCT	81 patients with compensated cirrhosis	G-CSF + CD133-positive cell infusion (*n* = 28), G-CSF (*n* = 26), SMT (*n* = 27)	No improvement in liver dysfunction or fibrosis	Serious adverse events were more frequent in the G-CSF and stem-cell infusion group, include ascites, sepsis and encephalopathy.
Philips, 2020 (65)/ retrospective study	100 patients with decompensated cirrhosis	G-CSF (*n* = 100), historical control (*n* = 24)	improvement in ascites at 3 months, unsustained improvement in CTP score, MELD score and encephalopathy	Higher mortality in G-CSF group. Serious adverse events include sepsis, variceal bleeds, encephalopathy, liver failure and liver cancer.

RCT, randomized controlled trial; UC-MSCs, human umbilical cord-derived mesenchymal stem cells; BM-MSCs, bone marrow mesenchymal stem cells; CTP, Child–Turcotte–Pugh; MSCs: mesenchymal stem cells; MELD, model for end stage liver disease; SMT, standard medical therapy; GH, growth hormone; TFS, transplant free survival; EPO, Erythropoietin.

## 6 Gut microbiota modulation: targeting the gut–liver axis

The human gut microbiota is a complex microecosystem, predominantly composed of Bacteroidetes, Firmicutes, Actinobacteria, and Proteobacteria, with functions encompassing nutritional metabolism, immunomodulation, and maintenance of intestinal barrier integrity. Under physiological conditions, the gut microbiota maintains a healthy intestinal barrier through multiple mechanisms, such as maintaining mucus, generating antimicrobial peptides, and producing short-chain fatty acids (SCFAs) (68).

There is a pathological interaction within the gut–liver axis. On the one hand, alterations in the intestinal environment caused by cirrhosis—such as hypoxia and hyperammonemia—induce dysbiosis of the gut microbiota. Research has demonstrated that the serum abundances of Clostridiales and Cyanobacteria levels are notably elevated in cirrhosis patients who have ascites compared to those who do not, accompanied by elevated levels of lipopolysaccharide-binding protein (LBP), suggesting increased bacterial translocation (69). According to Chen et al., patients with refractory ascites exhibited a gut microbiome landscape that was markedly shifted toward Gram-negative and potentially pathogenic species, alongside a marked decline in protective anaerobic taxa (70). On the other hand, gut microbiota dysbiosis leads to reduced production of SCFAs, resulting in diminished SCFA-mediated anti-inflammatory signaling. Moreover, it compromises intestinal barrier integrity and increases endotoxin translocation, which further activates inflammatory responses and exacerbates liver injury and the development of cirrhotic complications (8, 71, 72). What's more, the levels of SCFAs are inversely related to the severity of cirrhosis and are even lower in patients who have experienced ascites, hepatic encephalopathy, or spontaneous bacterial peritonitis (73).

Rifaximin, a non-absorbable antibiotic, can modulate the structure of the gut microbiota and decrease inflammatory cytokines like IF-10. It has been shown to decrease ascites volume (by a mean of 2.5 L), improve ascites-related symptoms, and enhance 6-month survival in patients with refractory ascites (74). In addition, low-dose rifaximin (800 mg/day) has been shown to decrease the occurrence of complications among patients with cirrhosis, including ascites aggravation and hepatic encephalopathy, and to improve survival in those with a Child-Pugh score ≥9 (75). These results imply that the gut microbiota could serve as a potential therapeutic target for managing ascites in cirrhosis, and that microbiota-based therapy holds potential as an emerging strategy for its management.

Several clinical trials have explored the potential benefits of probiotics for managing ascites in cirrhosis. In a single-blind, randomized, placebo-controlled study, Maslennikov et al. found that probiotic administration improved hyperdynamic circulation and Reduced complications related to cirrhosis, such as ascites and hepatic encephalopathy (76). VSL#3 is a multistrain probiotic formulation containing eight different bacterial species. Administering VSL#3 orally has been demonstrated to notably decrease portal hypertension, enhance circulatory function, and alleviate hypervolemia in patients in patients with cirrhosis and ascites (77). In addition, probiotic therapy with Saccharomyces boulardii has been shown to eliminate small intestinal bacterial overgrowth (SIBO), reduce systemic inflammatory responses, and decrease the incidence of ascites. Moreover, eradication of SIBO has been found to independently predict a favorable prognosis (78). Researches on mechanisms indicate that probiotics might enhance the function of the intestinal barrier through multiple mechanisms, including alleviating oxidative damage to the intestinal barrier, increasing the expression of tight junction proteins, and modulating the gut microbiota, thereby reducing bacterial translocation (79, 80).

Microbiota-based therapy offers several advantages for the management of ascites in cirrhosis, including a favorable safety profile and convenient oral administration, making it a promising approach. However, its efficacy exhibits considerable interindividual variability, and it is currently still in the early stages of clinical investigation. Large-scale randomized controlled trials are warranted to further validate its therapeutic efficacy for ascites in cirrhosis. Table 4 summarizes the potential benefits and risks of rifaximin and probiotics in clinical studies for treating ascites in cirrhosis.

**Table 4:** Clinical studies of rifaximin and probiotic for ascites in cirrhosis.

Study/ Type of study	Number and characteristics of study population	Study groups	Benefits	Risks
Lv, 2020 (74)/ RCT	75 cirrhotic patients with refractory ascites	rifaximin + intravenous antibiotics (*n* = 31), rifaximin (*n* = 19), SMT (*n* = 25)	Improvement in ascites control and the systemic inflammatory response; higher survival	No severe adverse events
Zeng, 2021 (75)/ multicentre, open-label RCT	200 patients with decompensated cirrhosis	rifaximin (*n* = 100), SMT (*n* = 100)	Improvement in ascites control and CTP score; decreased rate of hepatic encephalopathy and gastric variceal bleeding; prolonged liver transplantation-free survival in patients with poor liver function	No difference in the incidence of serious adverse events between the two groups
Maslennikov, 2022 (76)/ RCT	40 patients with Child-Pugh class B and C cirrhosis	probiotics (*n* = 24), placebo (*n* = 100)	Improvement in hyperdynamic circulation, systemic inflammation, liver function, ascites control, CTP score, and hepatic encephalopathy	No severe adverse events
Rincon, 2014 (77)/ single-arm trial	12 cirrhotic patients with ascites	VSL#3 probiotic (*n* = 12)	Improvement in hepatic and systemic haemodynamics and serum sodium levels	No severe adverse events
Efremova, 2024 (78)/ single-blind RCT	33 patients with Child-Pugh class B and C cirrhosis	probiotics (*n* = 20), placebo (*n* = 13)	Eradicated small intestinal bacterial overgrowth; improvement in ascites control, bacterial translocation, systemic inflammation and hyperdynamic circulation; decreased rate of hepatic encephalopathy	No severe adverse events

RCT, randomized controlled trial; SMT, standard medical therapy; CTP, Child–Turcotte–Pugh.

## 7 Limitations and future directions

Despite the promising results of novel therapies in current research on cirrhosis, the current evidence base remains weak. Most clinical studies on emerging therapies are conducted at a single center, with a small sample size and short follow-up, which restricts their ability to assess long-term efficacy and safety. Second, there is considerable heterogeneity across study populations. Factors such as the etiology of cirrhosis, Child-Pugh class, and the presence of other cirrhotic complications may all influence treatment outcomes; however, current studies lack adequate subgroup analyses, which limits the generalizability of the findings. Finally, refractory ascites is one of the most challenging complications of cirrhosis and is associated with a poor prognosis. Conventional treatment options remain suboptimal, making this population the most appropriate target for emerging therapies. However, very few studies have specifically focused on refractory ascites.

Future studies should include large-scale, multicenter clinical trials with long-term follow-up cohorts to evaluate long-term efficacy and safety. Furthermore, specific populations, such as patients with refractory ascites, should be considered as independent stratification groups or the primary enrollment population to better define treatment responses. Stratification strategies based on individual patient characteristics should be developed to facilitate personalized and precision medicine. In addition, combination therapy regimens involving different therapeutic modalities warrant further investigation.

## 8 Conclusion

With advances in medical care, the management of ascites in cirrhosis is evolving from traditional symptomatic treatment toward mechanism-guided, precision interventions. Emerging strategies including SGLT2 inhibitors, vasoconstrictors, regenerative medicine, and gut microbiota modulation, have shown promise in early-stage studies. However, large scale, multicenter clinical trials with long term follow-up are still needed to define their efficacy, safety, and optimal patient selection criteria in patients with ascites in cirrhosis. At present, these novel approaches should be explored within well monitored clinical research frameworks, serving as potential complements rather than replacements for standard therapy. It is anticipated that more precise and effective therapeutic options will become available in the future to improve the prognosis of patients with ascites in cirrhosis.
